# Chemical Composition and Antioxidant, Anti-Inflammatory, and Enzyme Inhibitory Activities of an Endemic Species from Southern Algeria: *Warionia saharae*

**DOI:** 10.3390/molecules26175257

**Published:** 2021-08-30

**Authors:** Habiba Rechek, Ammar Haouat, Kaouther Hamaidia, Hamza Allal, Tarek Boudiar, Diana C. G. A. Pinto, Susana M. Cardoso, Chawki Bensouici, Noureddine Soltani, Artur M. S. Silva

**Affiliations:** 1 Faculty of Sciences of Nature and Life, Mohamed Cherif Messaadia University, Souk-Ahras 41000, Algeria; habiba.rec@gmail.com; 2 Department of Biology of Organisms, Faculty of Sciences of Nature and Life, University of Batna 2, Mostefa Ben Boulaid, Batna 05078, Algeria; 3 LAQV-REQUIMTE & Department of Chemistry, University of Aveiro, 3810-193 Aveiro, Portugal; susanacardoso@ua.pt; 4 Unité de Valorisation des Ressources Naturelles, Molécules Bioactives et Analyse Physicochimiques et Biologiques (VARENBIOMOL), Université des Frères Mentouri, Constantine 25000, Algeria; haouatammar@gmail.com; 5 Department of Biology, Faculty of Sciences of Nature and Life, University of Oued Souf, Oued Souf 39000, Algeria; 6 Laboratory of Applied Animal Biology, Badji Mokhtar University, Annaba 23000, Algeria; noureddine.soltani@univ-annaba.org; 7 Department of Technology, Faculty of Technology, 20 August 1955 Skikda University, Skikda 21000, Algeria; hamzaallal07@gmail.com; 8 Centre de Recherche en Biotechnologie, Ali Mendjli Nouvelle Ville UV 03, Constantine 25000, Algeria; boudiar_tarek@yahoo.fr (T.B.); c.bensouici@crbt.dz (C.B.)

**Keywords:** *Warionia saharae*, UHPLC-DAD-ESI/MS, phenolic compounds, GC–MS analysis, antioxidant activity, anti-inflammatory activity, enzyme inhibition, molecular docking

## Abstract

*Warionia saharae* Benth. & Coss. (Asteraceae) is an endemic species of North Africa naturally grown in the southwest of the Algerian Sahara. In the present study, this species’ hydromethanolic leaf extract was investigated for its phenolic profile characterized by ultra-high-performance liquid chromatography coupled with a diode array detector and an electrospray mass spectrometer (UHPLC-DAD-ESI/MS). Additionally, the chemical composition of *W. saharae* was analyzed by gas chromatography–mass spectrometry, and its antioxidant potential was assessed through five in vitro tests: DPPH^●^ scavenging activity, ABTS^●+^ scavenging assay, galvinoxyl scavenging activity, ferric reducing power (FRP), and cupric reducing antioxidant capacity. The UHPLC-DAD-ESI/MS analysis allowed the detection and quantification of **22** compounds, with taxifolin as the dominant compound. The GC–MS analysis allowed the identification of **37** compounds, and the antioxidant activity data indicate that *W. saharae* extract has a very high capacity to capture radicals due to its richness in compounds with antioxidant capacity. The extract also showed potent α-glucosidase inhibition as well as a good anti-inflammatory activity. However, weak anti-α-amylase and anticholinesterase activities were recorded. Moreover, an in silico docking study was performed to highlight possible interactions between three significant compounds identified in *W. saharae* extract and α-glucosidase enzyme.

## 1. Introduction

It is recognized that medicinal plants have been used since the dawn of civilization to treat a wide range of ailments [1,2]. Their therapeutic potential has been attributed to a large quantity and variety of substances called secondary metabolites belonging to several classes such as phenolic compounds (e.g., flavonoids, chlorogenic acids) [3], terpenes [4], and alkaloids [5]. In parallel with increasing knowledge about their biological properties, plant secondary metabolites are increasingly used as drugs [6] and pharmaceutical food additives for aromatic and culinary purposes [7]. Various biotic and abiotic (light, temperature, soil water, soil fertility, and salinity) elicitors can influence their biosynthesis and accumulation [8,9,10].

Asteraceae is one of the most prominent plant families. It covers 23,000 species distributed across more than 1,620 genera. Among them, *Warionia saharae* Benth. & Coss. is an endemic species grown in Beni Ounif Zousfana and Bechar (south-west of Algerian Sahara). It was also found in various regions in the south of Morocco [11,12]. The leaves of *W. saharae* are used in folk medicine to treat many health problems, among them inflammatory diseases, gastrointestinal disorders, and epileptic crises [13,14].

*Warionia saharae*, until now, did not arouse researchers’ interest, and few works have been published about this species. These works were focused on different types of *W. saharae* extracts: aqueous extract [15], essential oil and ethyl acetate extract [16,17], and flavonoid-enriched extract [14]. Their antimicrobial [16,18], antioxidant [16,19], antidiabetic, and antihypertensive [14,20] activities were revealed. Moreover, only ten known compounds were isolated from *W. saharae* chloroform and ethyl acetate extracts and were identified using UV, IR, and NMR methods [19]. As far as we know, the UHPLC-ESI-MS and GC–MS compositions of *W. saharae* leaves have not been studied.

Furthermore, this species’ extracts and secondary metabolites remain less explored regarding their anti-inflammatory, cytotoxic, and enzyme inhibition activities. Therefore, our study will be the pioneer in this field. Simultaneously, evaluating the extracts’ antioxidant and anti-inflammatory potentials and their inhibition ability toward key enzymes involved in diabetes and neurodegenerative diseases (α-glucosidase, α-amylase, and acetylcholinesterase enzymes) was also an objective. Molecular docking experiments investigate the most energetically favorable interactions between bioactive compounds and enzymes [21]. In this context, molecular docking studies against α-glucosidase were conducted to unveil any possible interactions with the major phenolic compounds of *W. saharae* extract.

This research is expected to provide a better understanding of the chemical composition and the health benefits of this plant, which could contribute to new perspectives for developing new and potent pharmacological agents.

## 2. Results and Discussion

### 2.1. UHPLC-DAD-ESI-MS/MSn Characterization of W. saharae Extract

**Figure 1:**
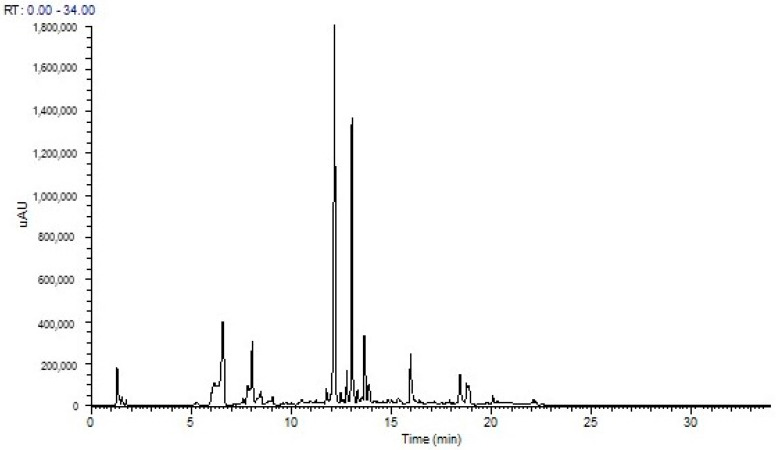
UHPLC chromatogram of *Warionia saharae* hydromethanolic extract recorded at 280 nm.

The UHPLC-DAD-ESI-MS/MS^n^ analysis of *W. saharae* hydromethanolic (80/20, *v*/*v*) extract allowed the identification, for the first time, of 24 compounds. Figure 1 shows the chromatographic profile of this extract, and in Table 1, with respect to their elution order, the peak characteristics, the assigned identification, and the quantification are detailed. The compounds’ assigned identification was enabled by comparing retention times and MS data from reference standards or comparing with previously reported MS and UV–Vis data.

Among the 24 metabolites, hydroxycinnamic acid derivatives and flavonoids were identified, which are secondary metabolites usually associated with anti-inflammatory properties. Actually, the quantification shows around 76 µg/mg extract of hydroxycinnamic acid derivatives and around 80 µg/mg extract of flavonoids (Table 1).

Considering the hydroxycinnamic acid derivatives, they are mostly chlorogenic acid derivatives, from which 1,3-*O*-dicaffeoylquinic acid (1,3-*O*-diCQA) can be highlighted because of its quantity (peak 18 at 13.03 min; Figure 1 and Table 1).

The other dicaffeoylquinic acids, isomers 3,5-*O*-diCQA, 3,4-*O*-diCQA, and 4,5-*O*-diCQA, were also identified, respectively, with peaks 11, 17, and 19. An extra dicaffeoylquinic acid was detected (peak 9, Table 1), but we could not establish its full assignment. These isomeric dicaffeoylquinic acids present a pseudomolecular ion [M-H]^−^ at *m/z* 515, and the characteristic fragments at *m/z* 353, 191, and 179, corresponding to monocaffeoyl quinic, quinic acid, and caffeic acid moieties, respectively [22,23,24]. Monocaffeoylquinic acids, compounds with a pseudomolecular ion [M-H]^−^ at *m/z* 353 and the typical MS2 fragment at *m/z* 191 (due to quinic acid), were also observed. Again, their identification was confirmed by literature data, and peaks 4, 5, and 6 were assigned to 5-*O*-CQA, 3-*O*-CQA, and 4-*O*-CQA, respectively [22].

The flavonoid family is represented in the hydromethanolic extract of *W. saharae* by 10 derivatives, 7 aglycones, and 3 glycosides. These results are consistent with those previously reported because we also found luteolin, esculetin, taxifolin, and quercetin derivatives, although Mezhoud et al. [19] mainly reported the flavonoids in their aglycone form.

Among the identified flavonoids, taxifolin derivatives can be highlighted (peaks 14 and 15, Table 1) with a pseudomolecular ion [M-H]^−^ at *m/z* 303, MS^2^ fragment ion at *m/z* 285, and UV spectrum data also in agreement with a taxifolin derivative [25,26,27]. Other flavonoid derivatives, such as luteolin derivatives (peaks 20, 21and 22, Table 1) and quercetin derivatives (peaks 10 and 13, Table 1), were identified using a pure standard [23,28] and based on MS^2^ fragmentation [29]. Isorhamnetin (peak 23) was identified using a standard and literature data, from which the MS^2^ fragment at *m/z* 300 obtained by losing the methyl group can be highlighted [30,31]. Finally, peak 16, with a pseudomolecular ion [M-H]^−^ at *m/z* 477 and MS^2^ fragment at *m/z* 315, which corresponds to the loss of a glycosyl group, was assigned to isorhamnetin-*O*-hexoside (Table 1).

Other compounds were identified, for example, peak 2 with a pseudomolecular ion [M-H]^−^ at *m/z* 137, characteristic of hydroxybenzoic acid [32], peak 3 with a pseudomolecular ion [M-H]^−^ at m/z 339 and a fragment ion at *m/z* 177 characteristic of esculetin-6-*O*-glucoside [33], and peak 24 with a pseudomolecular ion [M-H]^−^ at *m/z* 299 and predominant fragment ions at *m/z* 271 and 255 characteristic of naringenin derivatives (Table 1).

### 2.2. GC–MS Characterization of W. saharae Extract

**Figure 2:**
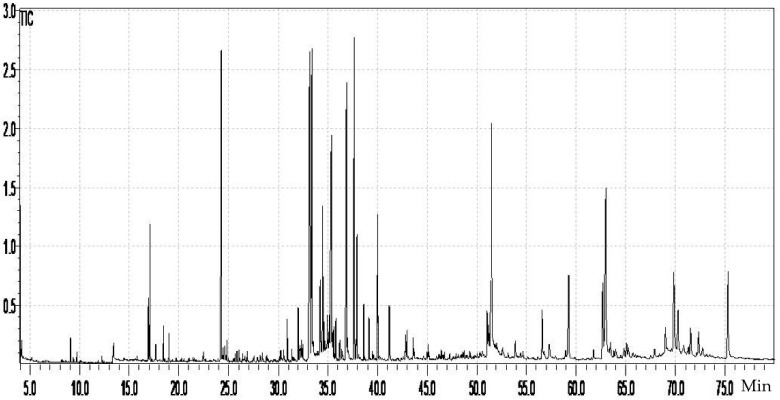
Total ion chromatogram (TIC) of *Warionia saharae* extract.

GC–MS analysis is the most suitable technique to assess plant extracts′ lipophilic profile [34,35]. It requires, in some cases, a derivatization procedure to convert many secondary metabolites to volatile derivatives [36], and silylation is the most recent derivatization procedure [37,38]. The GC–MS analysis of the hydromethanolic extract of *W. saharae* allowed the identification of four families of compounds: sugars (214.735 µg/mg), alcohols (128.520 µg/mg), carboxylic acids and esters (124.6 µg/mg), and fatty acids (11.364 µg/mg). The peak characteristics and the identification of the compounds are presented in Table 2, respecting their elution order. The quantification data were expressed as µg compound per mg of extract. The chromatogram is shown in Figure 2.

As far as we know, no studies have been carried out on the lipophilic composition of *W. saharae*, so this research provides new knowledge about this plant′s fatty acids and important alcohols (Table 2). Since we analyzed the hydromethanolic extract and, before its analysis by GC–MS, the extract was silylated, important chemical compounds such as sugars were revealed by the GC–MS analysis since they became lipophilic upon the silylation procedure. The sugars profile is, however, important to establish the plant nutritional value, and its richness in small carboxylic acids and alcohols also emphasizes the plant value. Moreover, they can be responsible for the observed activities discussed herein. Naturally, fatty acids represented the less significant group identified in the hydromethanolic extract of *W. saharae* because they are more lipophilic. Nevertheless, these compounds are known to benefit human health by decreasing cancer risk, cardiovascular and neurological disorders and through their antioxidant potential and anti-inflammatory activity [39,40].

### 2.3. Total Bioactive Content

Total phenolic and flavonoid contents in the hydromethanolic extract of *W. saharae* were found to be 119.8 ± 8.3 mg GAE/g of extract and 24.25 ± 0.070 mg QE/g extract, respectively. The most important properties of these compounds are their capacity to act as potent antioxidant [41], anti-inflammatory [42], anti-atherogenic [43], and anti-carcinogenic agents [44,45].

### 2.4. Antioxidant Activities

The antioxidant activity of *W. saharae* extract was evaluated through five in vitro tests: 1,1-diphenyl-2-picrylhydrazyl (DPPH^●^) scavenging activity, 2,2′-azino-bis(3-ethylbenzothiazoline-6-sulfonic acid (ABTS^●+^) scavenging assay, galvinoxyl^●^ scavenging assay, ferric reducing power (FRP), and cupric reducing antioxidant capacity (CUPRAC). The results obtained by the different tests, expressed in IC_50_ and A_0.50_, respectively, are shown in Table 3.

The antioxidant reaction with free radicals involves different mechanisms: the hydrogen atom transfer (HAT) method, in which the free radical removes one hydrogen atom from the antioxidant, and single electron transfer (SET), in which the antioxidant provides an electron to reduce compounds such as metals, carbonyls, and radicals. An antioxidant reaction can also involve both methods (HAT and SET) [46].

The use of a single assay could not assess the total antioxidant potential because of the implication of different mechanisms in the neutralization of free radicals [47]. Hence, to better assess the overall antioxidant effect, the *W. saharae* extract was assessed through five different trials (see Table 3).

According to ANOVA (F4,12 = 438.85, *p* < 0.001), data of DPPH^●^ scavenging activity show that the *W. saharae* hydromethanolic extract exhibited high antioxidant potential (IC_50_ = 7.12 ± 0.09 µg/mL) close to positive controls Trolox, BHT, and ascorbic acid, 5.12 ± 0.21, 6.14 ± 0.41, and 4.39 ± 0.01 µg/mL, respectively, but more potent than BHA (12.99 ± 0.41 µg/mL) with qTukey = 5.99.

According to the HSD Tukey test following ANOVA (F4,12 = 88.01, *p* < 0.001), in the ABTS^●+^ scavenging assay, there is not a significant difference between Trolox and ascorbic acid and between BHT and BHA (q_Tukey_ = 1.04 and 4.23, respectively). The studied extract exhibits high antioxidant activity (IC_50_ = 4.19 ± 0.35 µg/mL), very close to those of the standards.

This high antioxidant potential was also noted when analyzing the data of the galvinoxyl assay. Thus, *W. saharae* extract (IC_50_ = 3.55 ± 0.08 µg/mL) exhibits very high activity, statistically similar to both standards BHT and Trolox (F4,12 = 266.31, *p* < 0.001, qTukey = 1.38 and 4.65, respectively).

On the other hand, the reduction potential of the hydromethanolic extract of *W. saharae* was determined using FRP. In this assay, the antioxidant activity causes the reduction of the Fe^3+^/ferricyanid complex to the ferrous form. The FRP of extracts is linked with reductants, which exert antioxidant action by donating electrons and reacting with free radicals to convert them to more stable products [48,49]. A significant difference in the FRP assay was noted with ANOVA comparing all tests (F_4,12_ = 1446.84, *p* < 0.001, q_Tukey_ > 4.65). Data show that *W. saharae* extract activity (A0.5 = 31.21 ± 0.14 µg/mL) was weak compared to the used standards (5.25 ± 0.20, 7.99 ± 0.87, and 3.62 ± 0.29 µg/mL for Trolox, BHA, and ascorbic acid, respectively). Reducing capacity detected by cupric acid activity was significantly higher compared to that by the FRP assay.

The CUPRAC assay enables total antioxidant capacity (TAC) measurements of a plant extract using the copper (II)–neocuproine reagent in ammonium acetate buffer and based on an electron transfer mechanism [50]. According to the HSD Tukey test, all standards show statistically similar cupric acid activities (*p* > 0.05). A significant difference was noted with ANOVA and HSD Tukey tests (F_4, 12_ = 4.38, *p* < 0.026, q_Tukey_ < 4.65), which unveil the best activity of the *W. saharae* extract (A0.5 = 3.84 ± 0.16 μg/mL) as compared to all tested standards: Trolox, BHA, BHT, and ascorbic acid (8.69 ± 0.14, 6.62 ± 0.05, 8.97 ± 3.94, and 8.31 ± 0.15 µg/mL, respectively).

Overall, present data indicate that *W. saharae* extract showed a greater capacity to capture radicals. The results of the DPPH^●^ activity are in agreement with those reported in previous works on the ethyl acetate extract of the Algerian *W. saharae* (IC_50_ = 6.98 µg/mL) [19], but are significantly higher than those reported in another study conducted on the same species grown in Morocco (IC_50_ = 182 µg/mL) [16]. The nature of the extraction solvent, the plant growth area, and the collection period point out the discrepancy in antioxidant activity results between the referred studies. It should be mentioned that no previous studies were conducted on the antioxidant activity of *W. saharae* using the ABTS^●+^ scavenging assay, galvinoxyl assay, FRP, and cupric reducing antioxidant capacity.

The DPPH^●^, ABTS^●+^, and galvinoxyl scavenging data suggest that the components present in *W. saharae* extract act as excellent scavenging free radicals through a mechanism of electron or hydrogen donation to stabilize the free radicals, and should be able to protect biological matrices from free radical-mediated oxidative degradation [51,52].

According to the CUPRAC and FRP data, *W. saharae* was as effective as the positive standards in reducing Cu^2+^ to Cu^+^ but had a moderate effect in reducing the Fe^3+^ to Fe^2+^. The good reducing behavior of *W. saharae* extract suggests that its constituents, mainly chlorogenic acids, flavonoids, and sugars, are capable of terminating a chain reaction by eliminating free radical intermediates and hence are considered antioxidants [53]. A significant positive correlation between total phenolic and reducing sugar contents with the potent antioxidant capacity was established for other species [54]. Therefore, it is important to emphasize the important role of reducing sugars in antioxidant activity, since the extract of *W. saharae* contains a significant amount (around 215 μg/mg).

Furthermore, phenolic acids’ and flavonoids’ antioxidant potentials are closely related to their chemical structure [55] with double bonds and hydroxyl groups [56,57], making them ideal for scavenging free radicals and chelating metal ions. These properties also conferred effective antioxidant potential in vivo [58]. In the present study, the chemical composition analysis of the hydromethanolic extract of *W. saharae* reveals that phenolic acids are the most dominant group identified in this species. In addition, many compounds detected in the *W. saharae* hydromethanolic extract are known to possess great antioxidant capacity. For example, simple hydroxycinnamic acids have been reported to possess potent antioxidant activity against DPPH^●^ and ABTS^●+^ [59]. Chlorogenic acids are considered responsible for both green and medium roasted coffee’s antioxidant capacity, with 5-*O*-caffeoylquinic acid being the major contributor [60].

Li et al. [61] reported that di-*O*-caffeoylquinic acids exhibit high antioxidant activity using conventional antioxidant assays. The same authors proposed that their antioxidant mechanisms might include electron transfer, H^+^ transfer, and Fe^2+^ chelation.

Taxifolin (peak 14) is the major compound present in the hydromethanolic extract of *W. saharae* and may contribute to its strong antioxidant activity. A previous study reported that in antioxidant assays, taxifolin scavenges HO^●^, DPPH^●^, and ABTS^●+^ efficiently and enhances the relative Cu^2+^ and Fe^3+^ reducing levels [62]. Zu et al. [63] also mentioned that taxifolin is a free radical scavenger, and its antioxidant capacity is superior to other flavonoids. The results obtained by Topal et al. [64] from an in vitro study showed that taxifolin possesses marked antioxidant, reducing ability, radical scavenging, and metal-chelating activities.

Although it is present in lower amounts, luteolin has received increasing attention due to its various biological activities and pharmacological effects, including antioxidant activity [65], and has been widely applied in medicine and functional foods [66,67].

The high antioxidant potential recorded in this study may be linked to the presence of these compounds that could act individually or combined to provide the hydromethanolic extract of *W. saharae* with its strong antioxidant power.

### 2.5. Anti-Inflammatory Activity

The anti-inflammatory effect of *W. saharae* was evaluated by the NO^●^ scavenging method. NO^●^ is one of the main chemical mediators involved in inflammatory events. Regarding its ability to scavenge NO^●^, the relevant anti-inflammatory activity of *W. saharae* extract (IC_50_ = 76.66 ± 4.16 µg/mL) is better than that of ascorbic acid (IC_50_ = 144.380 ± 7.035 µg/mL) but lower than that of curcumin (IC_50_ = 7.49 ± 0.58 µg/mL). To the best of our knowledge, no anti-inflammatory effect had been previously described for *W. saharae* extract. Thus, this is the first report describing the anti-inflammatory potential of this species.

The phenolic compounds highly present in *W. saharae* extract are probably responsible for the anti-inflammatory property observed in this study, and flavonoids can be highlighted [42,68]. Taxifolin, the main phenolic compound present in *W. saharae* extract, was previously found to possess significant anti-inflammatory activity against carrageenan-induced edema involving the exudative phase of inflammation and also against formaldehyde-induced arthritis [69]. These results corroborate the traditional use of this species in folk medicine.

### 2.6. Enzyme Inhibitory Activities

The enzyme inhibitory activities of the *W. saharae* extract and the standard inhibitors tested toward α-glucosidase, α-amylase, and acetylcholinesterase expressed in IC_50_ or the percentage of inhibition are shown in Table 4.

The α-glucosidase and α-amylase inhibitors are used to treat diabetes mellitus as they slow down the digestion of carbohydrates in the small intestine and thus reduce the rapid rise in blood sugar after eating [70,71]. The inhibitory effect of the *W. saharae* species toward these two enzymes was evaluated in this study.

According to an ANOVA test (F1,5 = 349.79, *p* < 0.001), *W. saharae* extract expressed significant strong activity against the α-glucosidase enzyme with an IC_50_ = 23.52 ± 6.33 μg/mL lower than that of acarbose (IC_50_ = 405.77 ± 34.83 mg/mL). However, low inhibitory activity against α-amylase was recorded (38.41 ± 5.51% inhibition at the concentration of 4.2 mg/mL).

Several earlier studies have demonstrated the role of medicinal plants’ phenolic compounds as inhibitors of α-glucosidase. Among those flavonoids such as taxifolin and luteolin, and hydroxycinnamic acids, major compounds of *W. saharae* extract exhibited strong inhibitory activity against α-glucosidase in in vitro studies [72,73,74]. In a recent report, it was found that taxifolin′s pre-administration can significantly improve postprandial hyperglycemia in rats [74].

Furthermore, the obtained results with the α-amylase inhibition assay agree with those of Nyambe-Silavwe and Williamson [75] and Proença et al. [76], suggesting that chlorogenic and other phenolic acids, major compounds in *W. saharae* extract, weakly inhibit human α-amylase activity despite publications that claim otherwise [74].

The UHPLC-DAD-ESI/MS analysis shows that *W. saharae* extract contains these phenolic compounds abundantly. Consequently, it can be said that the synergistic effect could explain the high inhibitory effect recorded in the *W. saharae* extract among these compounds.

Apostolidis et al. [77] reported that medicinal plants that selectively inhibit α-glucosidase are better for controlling glucose absorption. Indeed, both enzymes’ inhibition leads to abnormal bacterial fermentation in the colon resulting from the undigested carbohydrates. Thus, the inhibition of α-glucosidase by the *W. saharae* extract could be considered a promising way to control diabetes mellitus.

Acetylcholinesterase inhibitors prevent the breakdown of acetylcholine in the body by blocking the action of the enzyme acetylcholinesterase responsible for its separation, which leads to an increase in its level in the space between nerve endings and thus gives it more opportunity to cross into another cell and carry out its functions. This mechanism is important for people with Alzheimer′s disease, who already suffer from a decrease in acetylcholine level since these inhibitors temporarily improve the symptoms of the disease and its stability [78].

We also evaluated the inhibitory effect of the acetylcholinesterase enzyme here. At the highest dose of the extract (1 mg/mL in this assay), the enzyme′s inhibition was observed (28.578 ± 2.979%). The inhibitory action on AChE in the in vitro assay proved to be lower than the donepezil used as a positive control (IC_50_ = 13.32 ± 1.34 µM). Thus, the weak inhibitory activity of *W. saharae* extract toward AChE activity could be related to the chemical composition of this species since we considered that the major substance in the plant extract is usually responsible for its biological activity.

Taxifolin, highly present in the *W. saharae* extract, has been shown to have relatively weak AChE inhibitory properties (IC_50_ = 133.1 μg/mL) [79] despite studies that claim otherwise [80]. Although chlorogenic acids are mainly responsible for inhibiting the AChE enzyme in green coffee extracts [81], their presence in *W. saharae* extract could not be sufficient to inhibit this enzyme strongly. Some of them, like 4,5-di-*O*-caffeoylquinic, one of the major compounds in *W. saharae* extract, showed AChE inhibition under 50% [81].

In addition, some phenolic compounds may act as activators of AChE. Earlier studies demonstrated that caffeic acid was shown to increase AChE activity in in vitro studies [82]. Some authors have hypothesized that caffeic acid (or its derivatives) could trigger the AChE catalytic site′s activation in a reaction at a second site [83]. Hence, the presence of caffeic acid in *W. saharae* extract could, in part, explain these results.

### 2.7. Molecular Docking

**Figure 3:**
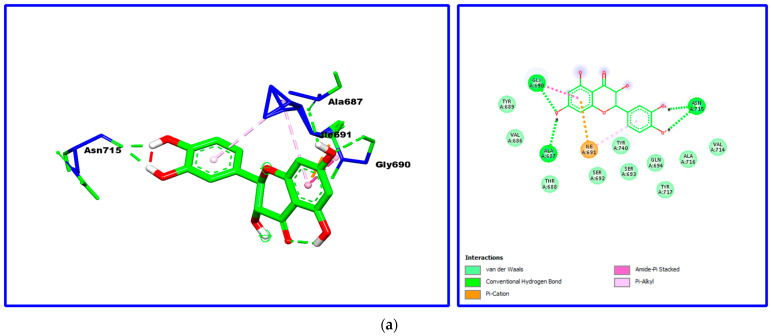

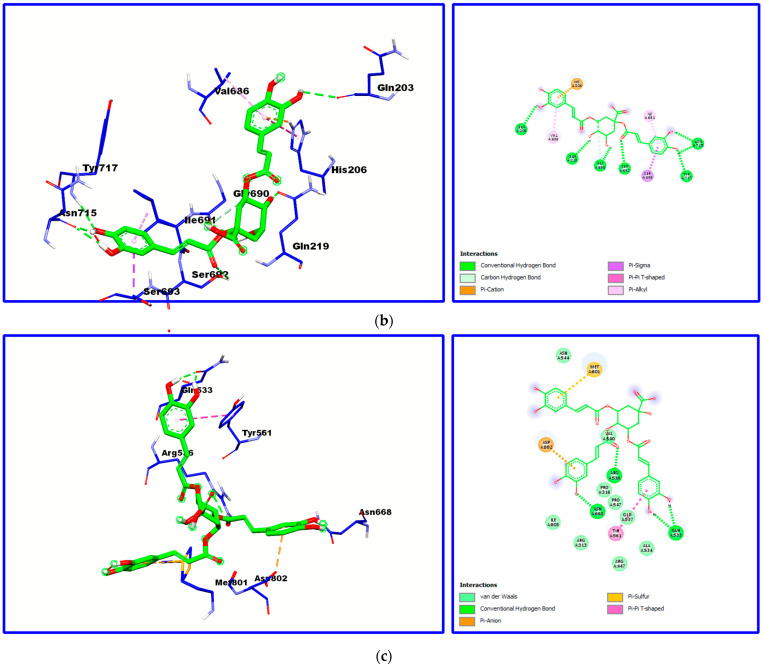
Two-dimensional and three-dimensional structures of the docked compounds taxifolin (**a**), 1,3-*O*-dicaffeoylquinic acid (**b**), and 4,5-*O*-dicaffeoylquinic acid (**c**) at the active site of α-glucosidase enzyme.

An in silico study was performed to further screen the mechanisms of interaction between α-glucosidase and the three major compounds in *W. saharae* extract. Figure 3 shows the 2D and 3D structures of the docked compounds at the active site of the α-glucosidase enzyme and the types of interactions involved.

Based on the docking test’s interaction data, the major constituents of *W. saharae* extract interact with α-glucosidase, engaging different amino acids (Table 5). Taxifolin showed a higher binding affinity, as indicated by its lower binding energy of –5.89 (kcal/mol), followed by 4,5-*O*-dicaffeoylquinic acid and 1,3-*O*-dicaffeoylquinic acid with a binding energy of –4.17 and –4.02 (kcal/mol), respectively.

Herein, it can be observed that the best docking pose obtained was for taxifolin docked to α-glucosidase. Different interactions, such as three hydrogen bonds with Asn715, Ala687, and Gly690, π–cation, and π–alkyl with Ile691 to the aromatic cycle and many other Van Der Waals interactions can be observed. Taxifolin, which has shown the best binding affinity within the major compounds, was previously reported as a competitive inhibitor of the α-glucosidase enzyme with an IC_50_ of 0.038 mg/mL [74].

Several types of interactions were found between both compounds, 4,5-*O*-dicaffeoylquinic acid and 1,3-*O*-dicaffeoylquinic acid, and the α-glucosidase active site (Figure 3), most of which are hydrogen bonds, π interactions, van der Waals, and carbon-hydrogen bonds.

These findings further support our in vitro assay results. However, in vivo studies are still necessary to better confirm these results.

## 3. Materials and Methods

### 3.1. Chemicals

The following chemicals: butylated hydroxyanisole (BHA), hydroxytoluene (BHT) and α-tocopherol, 1,1-diphenyl-2-picrylhydrazyl (DPPH^●^), 2,2′-azinobis(3-ethylbenzothiazoline-6-sulfonic acid) diammonium salt (ABTS^●+^), trichloroacetic acid (TCA), potassium ferricyanide, and neocuproine, were used for the determination of the antioxidant activities and were purchased from Sigma Chemical Co. (Sigma-Aldrich GmbH, Stern-heim, Germany). α-Glucosidase from Saccharomyces cerevisiae, 4-nitrophenyl α-D-glucopyranoside (pNPG), α-amylase from porcine pancreas, β-nicotinamide adenine dinucleotide (β-NADH), phenazine methosulphate (PMS), nitrotetrazolium blue chloride (NBT), and ascorbic acid were obtained from Sigma (St. Louis, MO, USA). Acarbose was purchased from Fluka (Bucharest, Romania) and potato starch from Fisher (Pittsburgh, PA, USA). Sodium nitroprusside, sulfanilamide, and 3,5-dinitrosalicylic acid (DNS) were obtained from Acros Organics (Hampton, NH, USA). Folin–Ciocalteu reagent, Na_2_CO_3_, and gallic acid were purchased from Panreac (Barcelona, Spain). Acetylcholinesterase (AChE) from Electrophorus electricus, 5,5-dithiobis(2-nitrobenzoic acid), acetylthiocholine iodine, and donepezil were purchased from Sigma-Aldrich (St. Louis, MO, USA).

Compounds used as standards to elucidate the identification of the phenolic constituents and to elaborate the calibration curves were obtained from EXTRASYNTHESE (Genay CEDEX, France).

Formic acid and acetonitrile HPLC-grade solvents were purchased from Panreac (Barcelona, Spain). Ultrapure water was obtained by a Direct-Q^®^ water purification system (Merck Life Science, Darmstadt, Germany). All other chemicals were of analytical grade.

Solvents used for GC–MS analysis were purchased from Panreac and Acros Organics and were of analytical grade. Other chemicals such as palmitic acid (99%), *N*,*O*-bis(trimethylsilyl)trifluoroacetamide (BSTFA) (99%), trimethylsilyl chloride (TMSCl) (99%), eicosane (99%), hexatriacontane (99%), citric acid (99.5%), cinnamic acid (99%), palmitic acid (99%), mannose (99%), D-mannitol (98%), 5-cholesten-3β-ol (99%), tetradecanol (98%), and 1-palmitoylglycerol (99%) were purchased from Sigma-Aldrich.

### 3.2. Extract Preparation

Aerial parts of *W. saharae* were collected from Djebel Antar (Bechar, Southwest Algeria; 31°56′34′′ N, 1°55′52′′ W). A voucher specimen was deposited in the Herbarium of the VAREN laboratory of Mentouri University of Constantine, Algeria, under the reference number VAREN/CWS04/05. The leaves were shade air-dried at room temperature (25 °C) before they were ground to a fine powder. The powder was then macerated at room temperature with a 80:20 mixture of methanol:water (*v*/*v*) for 72 h three times. The filtrates were concentrated under reduced pressure using a rotary evaporator at 30 °C. The residual water was freeze-dried in a freeze dryer and then stored at 4 °C until future use.

### 3.3. Total Bioactive Content

#### 3.3.1. Total Phenolic Content

Total phenolic content was determined using the Folin–Ciocalteu method [84]. An aliquot of 15 μL of plant extract (1 mg/mL) was mixed with 15 μL of Folin’s reagent and 60 μL of water. After 5 min of incubation, 150 μL of Na_2_CO_3_ solution (20% *w*/*v*) was added. The mixture was then incubated in the darkness for 60 min, and absorbance was read at 760 nm. The standard curve of gallic acid was obtained under the same conditions as above, and the total phenolic content was measured as μg equivalents of gallic acid per mg of extract (μg GAE mg of extract).

#### 3.3.2. Total Flavonoid Content

The total flavonoid content of the plant’s crude extract was determined according to the method described by Türkoğlu et al. [85] with a slight modification. A total of 100 µL of plant extract was mixed with 100 mL of AlCl_3_ (2%) solution. The mixture was incubated at room temperature for 10 min, and then the absorbance was measured at 415 nm. The standard curve of quercetin was obtained under the same conditions as above, and the total phenolic content was measured as μg equivalents of quercetin per mg of extract (μg GAE/mg of extract).

### 3.4. UHPLC-DAD-ESI-MS/MSn Characterization of W. saharae Extract

Phenolic composition of hydromethanolic extract of *W. saharae* was determined using an Ultimate 3000 (Dionex Co., San Jose, CA, USA) apparatus equipped with a binary pump, an automatic sampler, and a diode array detector (Dionex Co., San Jose, CA, USA). The MS analysis was performed using a Thermo LTQ XL mass spectrometer (Thermo Scientific, San Jose, CA, USA), which was equipped with an electrospray ionization interface (ESI). The separation was carried out with a Hypersil Gold (Thermo Scientific, USA) C18 column (100 mm length; 2.1 mm i.d.; 1.9 μm particle diameter, end-capped) at room temperature 25 °C. The extract was prepared at a 1 mg/mL concentration and the injection volume was 10 µL. The solvents used were formic acid in water (A) and acetonitrile (B). The flow rate was set at 2 mL/min. Spectra were recorded in negative mode. MS and MS/MS data were processed using the Thermo Xcalibur Qual Browser data system (Thermo Scientific, USA).

### 3.5. GC–MS Characterization of W. saharae Extract

The lipophilic composition of the hydromethanolic extract of *W. saharae* was analyzed by GC–MS using a Shimadzu Gas Chromatograph QP2010 Ultra. Three aliquots of the *W. saharae* extract (20 mg) were submitted to silylation procedure according to the method described by Freire et al. [86]. Silylation is the substitution of the active hydrogen atoms by a trimethylsilyl group (TMS), so that all the compounds with hydroxy groups will be transformed into the correspondent TMS derivatives. The sample was dissolved in 250 µL of dichloromethane. A total of 200 µL of hexatriacontane as internal standard (IS), 250 µL of pyridine, 250 µL of BSTFA, and 50 µL of TMSCl were then added to the sample. The mixtures were incubated for 30 min at 70 °C and then injected in triplicate into the GC–MS apparatus. Compound separation was carried out in J&W DB-5 capillary column (30 m × 0.25 mm inner diameter, 0.25 μm film thickness) using helium as the carrier gas (35 cm s^−1^). The following standards: eicontane, palmitic acid, tetradecanol, D-(+)-mannose, mannitol, and cholestanol, were used to elaborate calibration curves. Compounds were identified by comparing their mass spectra with mass spectra in the Wiley and NIST libraries and those available in the literature.

### 3.6. Antioxidant Activities

#### 3.6.1. Determination of 1,1-Diphenyl-2-Picrylhydrazyl Radical Scavenging Activity

The 1,1-diphenyl-2-picrylhydrazyl (DPPH^●^) free radical-scavenging activity was evaluated by a slightly modified method [87,88]. Briefly, the solution of 0.1 mM of DPPH^●^ was prepared in ethanol, and 160 µL of this solution was added to 40 µL of sample solutions diluted in methanol at different concentrations. Twenty-five minutes later, the absorbance was measured at 517 nm. The scavenging capability of DPPH^●^ was calculated using the following equation. The results were given as IC_50_ (mg/mL) corresponding to the concentration of 50% inhibition.

DPPH^●^ scavenging effect (%) = (A_C_ − A_S_)/A_C_ × 100

where Ac refers to the absorbance of control; A_S_ refers to the absorbance of sample.

The sample concentration providing 50% free radical scavenging activity (IC_50_) was calculated from the graph of DPPH^●^ scavenging effect percentage against sample concentration. BHA, BHT, Trolox, and ascorbic acid were used as antioxidant standards for comparison of the activity.

#### 3.6.2. Determination of 2,2′-Azinobis(3-Ethylbenzothiazoline-6-Sulfonic Acid) Scavenging Activity

The 2,2′-azino-bis(3-ethylbenzothiazoline-6-sulfonic acid) free cation-radical (ABTS^●+^) scavenging activity was obtained by spectrophotometric analysis according to the slightly modified method proposed by Re et al. [89]. The ABTS^●+^ was regenerated by adding 2.45 mM potassium persulfate to an aqueous solution of 7 mM ABTS^●+^, which was stored in the dark at room temperature for 12 h. Before use, the ABTS^●+^ solution was diluted until an absorbance of 0.700 ± 0.025 at 734 nm with ethanol was obtained. Then, to each well, in a 96-well plate, 160 µL of this solution and 40 µL of sample solutions diluted in methanol at different concentrations were added. After 10 min, the percentage inhibition at 734 nm was calculated for each concentration relative to a blank absorbance (methanol). The scavenging capability of ABTS^●+^ was calculated using the following equation:

ABTS^●+^ Scavenging effect (%) = (A_C_ − A_S_)/A_C_ × 100

where Ac refers to the absorbance of control; As refers to the absorbance of sample.

The sample concentration providing 50% cation radical scavenging activity (IC_50_) was calculated from the graph of ABTS^●+^ scavenging effect percentage against sample concentration. BHA, BHT, Trolox, and ascorbic acid were used as antioxidant standards for comparison of the activity.

#### 3.6.3. Galvinoxyl Radical Scavenging Activity

The trapping activity of the galvinoxyl radical was determined according to the method described by Shi et al. [90] in 96-well microplates. A total of 40 µL of the extract to be tested at different concentrations was placed in the wells of the microplate. A total of 160 µL of methanolic galvinoxyl solution (4 mg of galvinoxyl in 100 mL of methanol) was added to each well. The plate was then incubated in the dark for 2 h. Absorbance was measured at the wavelength of 428 nm.

A blank was prepared by mixing 160 µL of methanol with 40 µL of each extract. All tests were performed in triplicate. The percentage of inhibition of the galvinoxyl^●^ is expressed by the following formula:

Galvinoxyl^●^ Scavenging effect (%) = (Ac − As)/Ac × 100

where Ac refers to the absorbance of control; As refers to the absorbance of sample.

The concentration of the sample providing 50% inhibition (IC_50_) was calculated by relating the inhibition percentages to the concentrations of the extracts. The antioxidant capacity of the extract was compared with ascorbic acid, BHT, BHA, and Trolox used as antioxidant standards and prepared under the same conditions.

#### 3.6.4. Ferric Reducing Power Assay

The FRP assay was carried out according to the method of Oyaizu [91] with slight modification. Briefly, 40 μL of phosphate buffer (pH 6.6) and 50 μL of potassium ferricyanide (1%) (1 g of K_3_Fe(CN)_6_ in 100 mL H_2_O) were mixed with 10 μL plant extract at different concentrations and then incubated 20 min at 50 °C. A mixture of 50 mL trichloroacetic acid (TCA) (10%), 40 mL H_2_O, and 10 μL FeCl_3_ (0.1%) was then added. The absorbance of the reaction mixture was then recorded at 700 nm. Increased absorbance of the reaction mixture indicated increased FRP. The results were given as A_0.50_, which corresponds to the concentration producing 0.500 absorbance.

#### 3.6.5. Cupric Ion Reducing Antioxidant Capacity Assay

The cupric ion reducing antioxidant capacity (CUPRAC) assay was determined by the method described by Apak et al. [92]. A total of 40 µL of *W. saharae* extract at different concentrations was added to a solution composed of 50 µL Cu Cl_2_, 2H_2_O (10 mM), 50 µL neocuproine (7.5 mM), and 60 µL ammonium acetate buffer solution (1 M, pH 7.0). The reaction mixtures were incubated in the dark for 1 h. The absorbance was then measured at 450 nm.

The results were calculated as A_0.50_ (µg/mL) corresponding to the concentration indicating 0.500 of absorbance. The reduction capacity of the extracts was compared with that of BHA, BHT, Trolox, and ascorbic acid prepared under the same conditions.

### 3.7. Anti-Inflammatory Activity

The in vitro anti-inflammatory activity was determined by the ability of the extract to scavenge NO^•^ following the method described by Afonso et al. [93]. A total of 100 µL of each extract concentration was mixed with 100 µL of sodium nitroprusside (3.33 mM) solution prepared in PBS 100 mM (pH 7.4). After incubation for 10 min at room temperature under light irradiation, 100 µL of Griess reagent (equal volume of 1% of sulfanilamide and 0.1% of naphthyl ethylenediamine dihydrochloride in 2.5% of phosphoric acid) was then added to the mixture. The absorbance was measured at 562 nm in spectrophotometer. Curcumin was used as the reference compound.

The scavenging ability of NO^●^ was calculated using the following equation:

NO^●^ Scavenging effect (%) = (Ac − As)/Ac × 100

where Ac refers to the absorbance of control; As refers to the absorbance of sample.

The sample concentration providing 50% NO^●^ scavenging activity (IC_50_) was calculated from the graph of NO^●^ scavenging effect percentage against sample concentration.

### 3.8. Inhibition of Enzymatic Activities

#### 3.8.1. Inhibition of α-Glucosidase Activity

α-Glucosidase inhibition activity was measured by the method described by Neto et al. [94] with brief modifications. A total of 50 µL of each extract concentration was mixed with 50 µL of 4-nitrophenyl α-d-glucopyranoside (PNPG) solution used as substrate. A total of 100 µL of the α-glucosidase enzyme solution was then added to the mixture. The absorbance was measured at 405 nm at 37 °C, for 20 min every minute. Acarbose was used as reference.

The α-glucosidase inhibitory activity was expressed as percentage inhibition and was determined according to the following formula:

Inhibition % = (Ac − As)/Ac × 100

where Ac refers to the absorbance of control (enzyme and buffer); As refers to the absorbance of sample (enzyme and inhibitor).

IC values (concentration of inhibitor required to inhibit 50% of enzyme activity) were determined.

#### 3.8.2. Inhibition of α-Amylase Activity

α-Amylase inhibition activity was determined according to the method previously described by Wickramaratne et al. [95] with slight modifications. Briefly, 200 µL of each extract concentration was added to 400 µL of a 0.8% (*w*/*v*) starch solution dissolved in 20 mM phosphate buffer (pH 6.9, containing 6 mM of NaCl). After incubation at 37 °C for 5 min, the reaction was started with the addition of 200 µL of α-amylase solution. A total of 200 µL of the mixture was then mixed with 600 µL of 3,5-dinitrosalicylic acid (DNS) reagent.

A second aliquot of 200 µL of the first mixture was also collected 15 min later and mixed with DNS reagent. The mixtures were boiled for 10 min. After cooling, 250 µL was transferred to each well in a 96-well microplate and the absorbance was read at 450 nm.

The α-amylase inhibitory effect was calculated by the following equation:

Inhibition % = (Ac − As)/Ac × 100

where Ac refers to the absorbance of control (enzyme and buffer); As refers to the absorbance of sample (enzyme and inhibitor).

IC values (concentration of inhibitor required to inhibit 50% of enzyme activity) were determined. Acarbose was used as reference compound.

#### 3.8.3. Inhibition of Acetylcholinesterase Activity

Inhibition of acetylcholinesterase (AChE) activity was determined as described by Ellman et al. [96] with slight modifications. A total of 150 µL of 0.025 U/mL solution of AChE was added to 50 µL of each extract at different concentrations. After incubating at 37 °C for 5 min, 50 µL of DTNB (0.5 mM) and 50 µL of acetylthiocholine iodide (2.5 mM) were added to the mixture. The absorbance was read at 415 nm each 150 s for 7.5 min. Donepezil was used as a reference. The percentage inhibition of AChE enzyme was determined according to the following formula:

Percentage of inhibition = (E − S)/E × 100.

where E is the activity of the enzyme without extract; S is the activity of the enzyme with the extract.

IC_50_ values (concentration of inhibitor required to inhibit 50% of enzyme activity) were determined.

### 3.9. Molecular Docking Study

To support our findings from in vitro inhibition of α-glucosidase activity by the *W. saharae* extract, three compounds were docked to the α-glucosidase enzymatic activity. The selected compounds were taxifolin, 1,3-*O*-dicaffeoylquinic acid, and 4,5-*O*-dicaffeoylquinic acid, which are the most abundant in the *W. saharae* extract. The 3D structures of the considered molecules were downloaded from PubChem database [97]. All three structures were performed by AM1 [H2] semi empirical method by using Orca-4.2.1 program package [98]. The crystal structure of the α-glucosidase (PDB code 3W37) was retrieved from the Protein Data Bank [99]. Water molecules were removed from the protein structure. The docking software AutoGrid and AutoDock (version 4.2.6) implemented in AutoDock Tools (ADT 1.5.6) program were used for the docking experiments. The most stable receptor–ligand complexes were generated using Chimera [100] and BIOVIA Discovery studio visualizer (version 1.10.2) [101].

### 3.10. Statistical Analysis

All of the data were analyzed with MINITAB software (version 16, State College, PA, USA) using one-way ANOVA followed by a post hoc honestly significant difference (HSD) Tukey’s test at *p* ≤ 0.05. Data were recorded as the mean ± standard deviation (m ± SD) from three independent assays.

## 4. Conclusions

Our results provided new information and allowed the characterization of the chemical profile of the hydromethanolic extract of *W. saharae*. The extract showed potent antioxidant and α-glucosidase inhibition properties, highlighting that this species provides a promising source for obtaining natural compounds that could be used to develop food additives or pharmaceutical products. Anti-inflammatory properties were also observed with the hydromethanolic extract of *W. saharae*. Finally, further toxicological and pharmacological studies must be performed for pharmaceutical purposes.

## Figures and Tables

**Table 1:** Chemical composition of *W. saharae* extract by UHPLC-DAD-ESI/MS (retention time (RT), not quantified (NQ), traces (Tr), caffeoylquinic acid (CQA)).

N°	RT	Λ_max_	[M − H]^−^	MS^2^ (*m/z*)	Identified Compound	Quantification (µg/mg Extract)
**1**	1.53	256	215	179,160	Caffeic acid adduct with chloride	NQ
**2**	1.75	194	137	110,82	Hydroxybenzoic acid	0.855 ± 0.037
**3**	6.58	204,224,250,289,340	339	177	Esculetin-6-*O*-glucoside	NQ
**4**	8.05	325,238,218	353	191,179	5-*O*-CQA	10.333 ± 2.360
**5**	8.29	203,225,337	353	191,179	3-*O*-CQA	1.537 ± 0.239
**6**	8.46	202,225,298,342	353	191,179,173,135	4-*O*-CQA	3.039 ± 0.420
**7**	8.84	299,235,322	179	135	Caffeic acid	1.380 ± 0.790
**8**	9.06	260	536	-	Unknown	NQ
**9**	10.54	198,229,288	515	353	*O*-diCQA isomer	1.631 ± 0.430
**10**	10.96	234,288,330	477	301	Quercetin glucuronide	0.560 ± 0.110
**11**	11.23	232,290	515	353,335,179	3,5-*O*-diCQA	1.280 ± 0.190
**12**	11.76	261	609	459,315,299	Unknown	NQ
**13**	12.03	204,256,353	463	301,300	Quercetin-*O*-hexoside	2.460 ± 0.790
**14**	12.15	227,289	303	285,175,125	Taxifolin	62.800 ± 16.690
**15**	12.47	231,290	303	285,177	Taxifolin isomer	1.540 ± 0.590
**16**	12.60	236,256,268,341	477	315,300	Isorhamnetin-*O*-hexoside	0.640 ± 0.130
**17**	12.78	220,241,324	515	353,335,172,191	3,4-*O*-diCQA	4.185 ± 1.340
**18**	13.03	220,241,324	515	353,335,191	1,3-*O*-diCQA	43.120 ± 17.390
**19**	13.67	219,242,326	515	353,299,255,203	4,5-*O*-diCQA	9.660 ± 2.970
**20**	15.97	207,254,351	285	241,217,199,175	Luteolin	7.410 ± 0.530
**21**	17.91	245,274,332	299	284	3′-*O*-Methylluteolin (Chrysoeriol)	0.740 ± 0.520
**22**	18.07	246,342	299	284	Luteolin 4′-methyl ether (Diosmetin)	Tr
**23**	18.43	296,234	315	300	Isorhamnetin	3.435 ± 1.390
**24**	18.76	200,230,290	299	271,255,243	Naringenin derivative	NQ

**Table 2:** Identified compounds on the hydromethanolic extract of *W. saharae* aerial parts by GC–MS (µg compound/mg extract). (SD: standard deviation, Rt: retention time, NQ: not quantified, Tr: traces).

Families	Peak	Rt (min)	Identified Compound *	Quantification
Carboxylic acids and esters Total=124.6 µg/mg	4	24.259	Malic acid	50.79 ± 5.67
	11	33.568	Citric acid	4.01 ± 1.98
	13	34.517	Quinic acid	18.53 ± 2.19
	14	34.960	2-*n*-Propyl valerate	4.63 ± 0.47
	23	37.945	D-Gluconic acid	17.11 ± 1.37
	28	41.191	Caffeic acid	6.02 ± 0.72
	37	62.690	Chlorogenic acid 1	19.89 ± 2.89
	39	65.073	Chlorogenic acid 2	3.62 ± 0.77
Fatty acids Total=11.364 µg/mg	3	18.452	Butanedioic acid	3.58 ± 0.56
	25	39.147	Palmitic acid	5.73 ± 0.12
	29	42.814	Linoelaidic acid	1.056 ± 0.80
	30	42.961	Oleic acid	0.98 ± 0.11
Alcohols Total=128.520 µg/mg	2	17.099	Glycerol	12.03 ± 2.07
	5	30.892	D-arabitol	2.17 ± 0.66
	16	35.335	Dulcitol	31.99 ± 1.93
	21	36.916	*Myo*-inositol	60.91 ± 6.91
	24	38.630	*Scyllo*-Inositol	4.24 ± 0.90
	26	40.015	*Myo*-Inositol	17.18 ± 3.16
Sugars Total=214.735 µg/mg	6	32.041	Ribonic acid	Tr
	7	32.222	D-Talose	Tr
	8	32.379	D-Tagatose	Tr
	9	33.230	D-Fructose (isomer 1) ^a^	51.21 ± 14.36
	10	33.442	D-Fructose (isomer 2) ^a^	32.19 ± 19.31
	12	34.249	D-Galactose (isomer 1) ^b^	2.18 ± 0.14
	17	35.393	D-Galactose (isomer 2) ^b^	34.57 ± 0.46
	18	35.615	D-Galactose (isomer 3) ^b^	Tr
	22	37.677	D-Galactose (isomer 4) ^b^	19.81 ± 4.66
	31	51.024	D-Fructose (isomer 3) ^a^	Tr
	32	51.153	D-Turanose	Tr
	33	51.515	Sucrose derivative	36.27 ± 12.46
	34	53.916	D-Trehalose	Tr
	35	56.602	β-Lyxose	0.075 ± 0.01
	38	62.999	D-Glucose (isomer 1) ^c^	26.06 ± 4.58
	40	68.978	D-Gulose	Tr
	41	69.824	Sucrose	12.37 ± 0.03
	43	71.578	D-Glucose (isomer 2) ^c^	Tr
	44	72.381	D-Glucose (isómer 3) ^c^	Tr
Other compounds	1	16.930	Phosphoric acid	NQ
	15	35.172	Gluconolactone	NQ
	36	59.258	Catechin	NQ
	27	40.082	Esculetin	NQ
	19	35.829	Acrylsaeure	NQ
	42	70.226	5-Methyluridine	NQ

* Compounds were identified by comparison with the GC–MS spectral libraries NIST14.lib and WILEY229.LIB; ^a^ Different forms of D-fructose that can be obtained during the silylation; ^b^ Different forms of D-galactose that can be obtained during the silylation; ^c^ Different forms of D-glucose that can be obtained during the silylation.

**Table 3:** Antioxidant activity of the hydromethanolic extract of *W. saharae* by DPPH^●^, ABTS^●+^, galvinoxyl^●^, FRP, and CUPRAC assays.

Sample	Radical Scavenging Activity IC_50_ (μg/mL)			Radical Scavenging Activity A_0.5_ (μg/mL)	
	DPPH^●^	ABTS^●+^	Galvinoxyl	FRP	CUPRAC
*W. saharae*	7.12 ± 0.09	4.19 ± 0.35	3.55 ± 0.08	31.21 ± 0.14	3.84 ± 0.16
Trolox	5.12 ± 0.21	3.21 ± 0.06	4.31 ± 0.05	5.25 ± 0.20	8.69 ± 0.14
BHA	6.14 ± 0.41	1.81 ± 0.10	5.38 ± 0.06	7.99 ± 0.87	6.62 ± 0.05
BHT	12.99 ± 0.41	1.29 ± 0.30	3.32 ± 0.18	>200	8.97 ± 3.94
Ascorbic acid	4.39 ± 0.01	3.04 ± 0.05	5.02 ± 0.01	3.62 ± 0.29	8.31 ± 0.15

IC_50_ and A_0.50_ values corresponded to the concentration of 50% inhibition percentages and the concentration at 0.500 absorbance, respectively. For the same test, different small letters in each column are statistically different at *p* ≤ 0.05 (HSD Tukey test). DPPH^●^: 1,1-diphenyl-2-picrylhydrazyl, ABTS^●+^: 2,2′-azino-bis(3-ethylbenzothiazoline-6-sulfonic acid, FRP: ferric reducing power, CUPRAC: cupric ion reducing antioxidant capacity, Trolox: 6-hydroxy-2,5,7,8-tetramethylchroman-2-carboxylic acid, BHA: butylated hydroxyanisole, BHT: hydroxytoluene (BHT).

**Table 4:** Enzyme inhibition activities of *W. saharae* extract and standards (IC_50_ value corresponded to the concentration of 50% inhibition.

Sample	Enzyme Inhibitory Activity		
	α-Glucosidase IC_50_ (μg/mL)	α-Amylase (% inhibition)	Acetylcholinesterase (% inhibition)
*W. saharae* extract	23.52 ± 6.33 ^A^	38.41 ± 5.51 ^a^	28.578 ± 2.979 ^b^
Acarbose (μg/mL)	405.77 ± 34.83 ^B^	ND	ND

For the same enzyme, different capital letters in each column are statistically different at *p* ≤ 0.05 (HSD Tukey test).^a^ Determined at the concentration of 4.2 mg/mL of the extract. ^b^ Determined at the concentration of 1 mg/mL of the extract.

**Table 5:** Binding energy and amino acids involved in the interactions between α-glucosidase enzyme and the docked compounds.

Compound	BE	Amino Acid Involved in Interaction
Taxifolin	−5.89	Asn715, Ile691, Ala687, Gly690.
4,5-*O*-Dicaffeoylquinic acid	−4.02	His206, Ile691, Asn415, Tyr717, Ser693, Ser692, Gly690, Gln219, Val686, Gln208.
1,3-*O*-Dicaffeoylquinic acid	−4.17	Met801, Gln533, Tyr561, Arg536, Asn668, Asp802.

BE: Binding energy (kcal/mol).

## Data Availability

Data is contained within the article.

## Sample Availability

Samples of the compounds are available from the authors.
